# HIV-1 Nef alters podosomes and promotes the mesenchymal migration in human macrophages

**DOI:** 10.1186/1742-4690-10-S1-P96

**Published:** 2013-09-19

**Authors:** Christel Vérollet, Emilie Bonnaud, Cassandre Kinnaer, Renaud Poincloux, Annabelle Corjeon, Isabelle Maridonneau-Parini

**Affiliations:** 1 CNRS UMR 5089; IPBS (Institut de Pharmacologic et de Biologie Structurale), BP64182, 205 Route de Narbonne, 31077 Toulouse cedex 04, France; 2 Université de Toulouse, UPS; IPBS; F-31077, France

## Background

Macrophages are a cell target of Human Immunodeficiency Virus-1 (HIV-1). They play a key role in AIDS pathogenesis as long-term viral reservoirs. As macrophages are able to migrate in all body tissues, they are also susceptible to participating in the systemic dissemination of viruses.

## Results

Here, we examined the migration ability of HIV-1-infected primary human macrophages. We show that HIV-1 infection modifies dramatically the migration of macrophages in 3-dimentionnal (3D) environments. While the amoeboid migration mode is inhibited upon infection, another migration mode specifically used by macrophages in dense 3D environments, the mesenchymal migration mode, is greatly enhanced. HIV-1 negative factor (Nef) is responsible for both effects on macrophage migration modes. Consistently with an increase in mesenchymal migration capacities, Nef accumulates around F-actin structures necessary for proteolysis of the extracellular matrix, e.g. podosomes, and alters their structure, function and dynamics.

Mechanistically, HIV-1-induced podosome modifications and mesenchymal macrophage migration depend on Nef’s ability to activate the macrophage-specific Src tyrosine kinase, Hck.

## Conclusions

We conclude that HIV-1, through the action of Nef, is able to force macrophages to preferentially infiltrate some tissues. Thus, interfering with Nef/Hck interaction emerges as an unexpected strategy to reduce the spread of the virus by macrophages, for example in the brain of patients where the presence of infected macrophages is associated with neurotoxicity and AIDS-associated dementia.

